# Clinicopathological and Prognostic Significance of Stromal Patterns in Oral Squamous Cell Carcinoma

**DOI:** 10.3389/fmed.2022.859144

**Published:** 2022-04-15

**Authors:** Yusuke Amano, Atsushi Kihara, Masayo Hasegawa, Tamaki Miura, Daisuke Matsubara, Noriyoshi Fukushima, Hiroshi Nishino, Yoshiyuki Mori, Toshiro Niki

**Affiliations:** ^1^Department of Integrative Pathology, Jichi Medical University, Shimotsuke, Japan; ^2^Department of Otolaryngology, Jichi Medical University, Shimotsuke, Japan; ^3^Department of Dentistry, Oral and Maxillofacial Surgery, Jichi Medical University, Shimotsuke, Japan

**Keywords:** oral squamous cell carcinoma, desmoplastic reaction, stromal pattern, prognosis, epithelial-mesenchymal transition

## Abstract

**Background:**

Stromal patterns (SP), especially desmoplastic reactions, have recently gained attention as indicators of malignant potential in cancer. In this study, we explored the clinicopathological and prognostic significance of the SP in oral squamous cell carcinoma (OSCC).

**Materials and Methods:**

We reviewed 232 cases of surgically resected OSCC that were not treated with neoadjuvant chemoradiotherapy. We categorized the SP of the OSCC into four groups: immune/inflammatory (84 cases), mature (14 cases), intermediate (78 cases), or immature (56 cases).

**Results:**

The SP category was significantly associated with various clinicopathological factors, such as the histological grade, lymphovascular invasion, neural invasion, and a diffuse invasion pattern. For each of the factors, the immune/inflammatory type was associated with favorable categories, while the immature type was associated with unfavorable categories (p ≤ 0.001). The SP category was also shown to be a prognostic predictor: the 5-year relapse-free survival (RFS) rate was 72.0% for the immune/inflammatory type, 66.7% for the intermediate/mature type, and 31.2% for the immature type (*p* < 0.0001), and the 5-year overall survival (OS) rate was 85.1% for the immune/inflammatory type, 76.4% for the intermediate/mature type, and 50.0% for the immature type (*p* < 0.0001). In multivariate analyses, the SP category was identified as an independent prognostic factor for RFS and OS.

**Conclusion:**

Our SP categorization method provides valuable prognostic information in OSCC.

## Introduction

Oral squamous cell carcinoma (OSCC) is one of the most common types of malignant head and neck cancer (1). Generally, patients with head and neck squamous cell carcinoma (HNSCC), including OSCC, have advanced disease at the time of the initial diagnosis (2). Despite the advent of chemotherapy, radiotherapy, and multimodal therapy, the 5-year overall survival (OS) rate of HNSCC has remained approximately 50% for the past 30 years (3). Currently, there are no reliable clinical biomarkers that can be used to stratify HNSCC patients according to their prognosis (3).

Treatment planning for OSCC is generally based on disease staging; i.e., clinical assessment of the tumor, lymph nodes, and distant metastases (TNM staging) (4). Histopathological prognostic markers are mainly associated with the properties of cancer cells (e.g., the degree of differentiation, nuclear pleomorphism, and mitotic activity). However, tumor growth also depends on the stroma surrounding the tumor and the tumor microenvironment (TME), which plays an important role in cancer progression (5). The tumor stroma basically consists of the non-malignant cells in the TME, including cancer-associated fibroblasts (CAF), innate and adaptive immune cells, microvessels, and extracellular matrix (ECM) cells (6). The tumor stroma is formed by a complex process induced by tumor-host interactions, and studies have shown that the histological features of the tumor stroma may serve as independent prognostic factors in various solid tumors (7).

Although several studies have reported the prognostic value of stromal features in OSCC, such as the tumor-stromal ratio (8, 9) and the number of CAF (alpha-smooth muscle actin-positive fibroblasts) (10) no previous studies have categorized stromal pattern (SP) by analyzing both stromal desmoplasia and immune/inflammatory cell reactions in an integrated manner.

Here, we propose a new method for categorizing SP in OSCC. This categorization system is largely based on Ueno's criteria (11), but we modified it by adding a fourth category; i.e., the immune/inflammatory type. This modification was made because we observed that a subset of OSCC showed characteristic immune/inflammatory cell stromal reactions in the absence of marked desmoplasia. We show here that the categories in this new system are closely associated with various clinicopathological parameters, such as the histological grade, lymphovascular invasion, neural invasion, and a diffuse invasion pattern, and represent an independent prognostic factor for OSCC.

## Materials and Methods

### Patients and Tumors

The study protocol was approved by the ethics committee of Jichi Medical University (Approval ID: A19-226). Tumor specimens were obtained from 232 patients who underwent head and neck cancer surgery without neoadjuvant chemoradiotherapy at Jichi Medical University Hospital between 2010 and 2018 for squamous cell carcinoma (SCC). Small specimens obtained by biopsy were not included in this study. The tumors were diagnosed and classified according to the World Health Organization (WHO) 2017 criteria (12). Information regarding the following factors was collected for each case: age, sex, the primary tumor site, the histological grade of the tumor, the tumor stage, lymph node metastasis, the mode of invasion according to the Yamamoto-Kohama (YK) classification (13), lymphovascular invasion, perineural invasion, methods of surgical reconstruction, lymph node dissection, postoperative treatments, and patient survival. The patient cohort consisted of 127 males and 105 females, who ranged in age from 29 to 92 years (mean age: 64.3 years). The primary tumor sites were as follows: the tongue (*n* = 144), the gingiva (*n* = 53), the buccal mucosa (*n* = 14), and the oral floor (*n* = 21). The pathological stage was defined according to the TNM classification established by the American Joint Committee on Cancer (AJCC) and the International Union Against Cancer (UICC) (4). The morphological pattern of the invasive front of carcinoma was also defined based on the YK classification: grade 1, well-defined borders; grade 2, less prominent borders; grade 3, group of cells and no distinct borders; grade 4C, diffuse invasion of the cord-like type; and grade 4D, diffuse invasion of the widespread type (13). The duration of the follow-up period ranged from 37–3,441 days (median: 1,277 days).

### Histological Classification of Stromal Patterns

**Figure 1:**
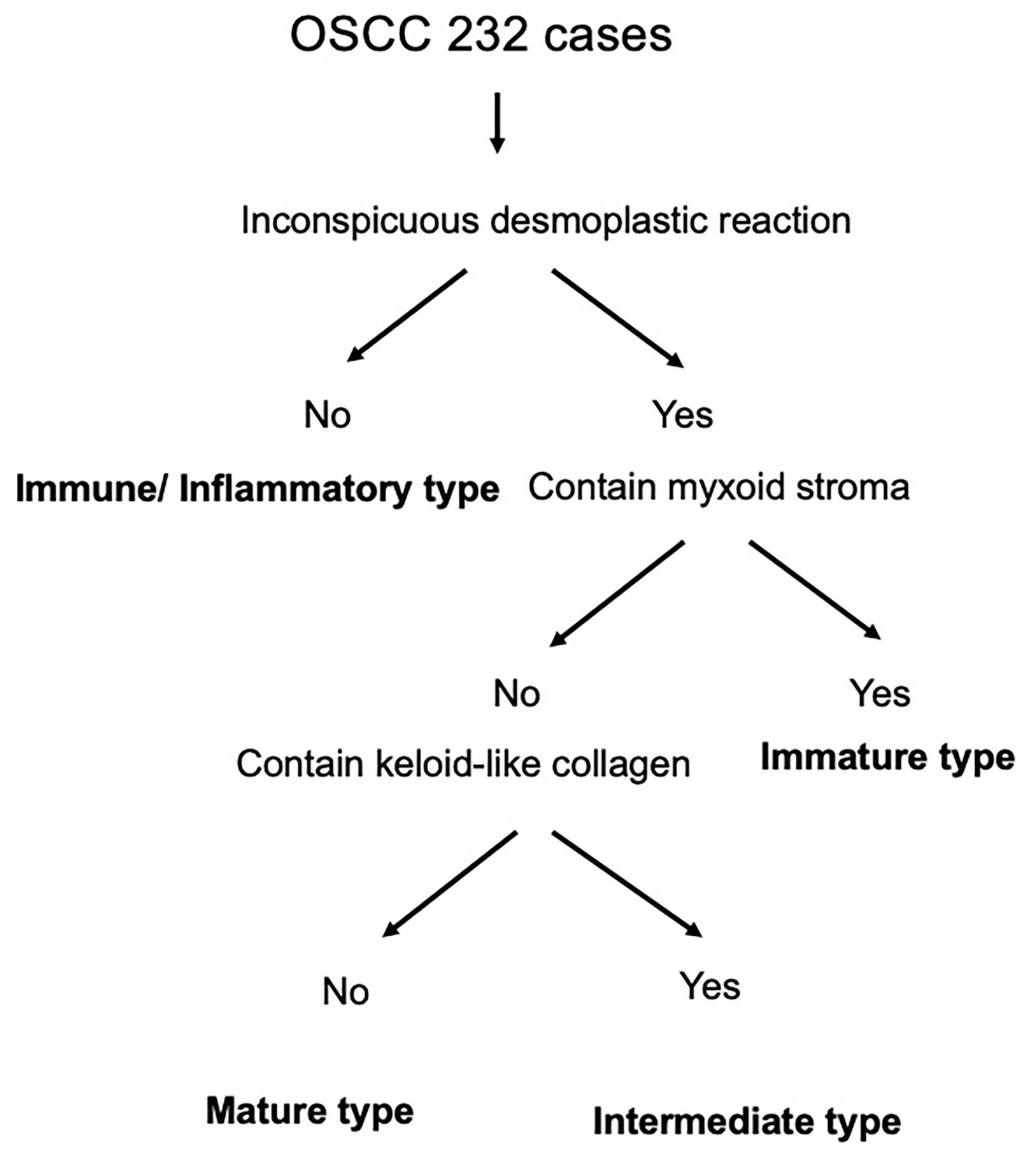
Flow chart of the stromal pattern (SP) categorization process.

The SP were categorized into four groups based on the presence/absence of a desmoplastic reaction (DR), a diffuse immune/inflammatory reaction, keloid-like collagen, and/or myxoid stroma at the invasive front (Figure 1).

To assess the SP, we first selected the sections representing the deepest invasive part of the primary tumors. Then, we scanned the whole of the HE-stained section at low magnification to determine the presence/absence of a DR or diffuse immune/inflammatory reaction at the invasive front. In this initial step, the immune/inflammatory type, which involved diffuse immune/inflammatory cell infiltration and an inconspicuous DR, was identified. Then, we examined any areas that exhibited DR at higher magnification to determine whether a myxoid stroma or keloid-like collagen was present. A myxoid stroma was defined as an amorphous stromal substance comprised of an amphophilic or slightly basophilic ECM, usually intermingled with randomly oriented hyalinized collagen molecules (11). Keloid-like collagen was defined as hyalinized thick bundles of hypocellular collagen that exhibited bright eosinophilic hyalinization, as is typically observed in keloids (11).

Thus, after identifying the immune/inflammatory type, the remaining cases were divided into immature, intermediate, and mature types, depending on the presence/absence of a myxoid stroma or keloid-like collagen in a desmoplastic stroma (Figure 1). As for the minimum amount of myxoid stroma required to classify a lesion as belonging to the immature type, the microscopic field of a ×40 objective lens was used (11). The stroma around ulcers and areas of necrosis were excluded from consideration.

All cases were examined independently by two pathologists (YA and MH), who were blinded to the clinical information. In cases in which the evaluations of the two pathologists differed, agreement was reached by consensus using a two-headed microscope.

### Statistical Analyses

The associations between clinicopathological factors and each SP category were analyzed using the chi-squared test. Relapse-free survival (RFS) and overall survival (OS) rates were analyzed according to the Kaplan-Meier method, and differences between groups were calculated using the log-rank test. Univariate and multivariate analyses were carried out using the Cox proportional hazards model, and hazard ratios (HR) with 95% confidence intervals (95% CI) and *p*-values were calculated. We first performed univariate analysis for each conventional clinicopathologic factors and the SP pattern. We then chose the factors that showed statistical significance, and used them in multivariate analysis. *p* < 0.05 were considered statistically significant. All statistical analyses were performed using the statistical software BellCurve for Excel (Social Survey Research Information Co., Ltd.).

## Results

### Clinicopathological Characterization of SP

**Figure 2:**
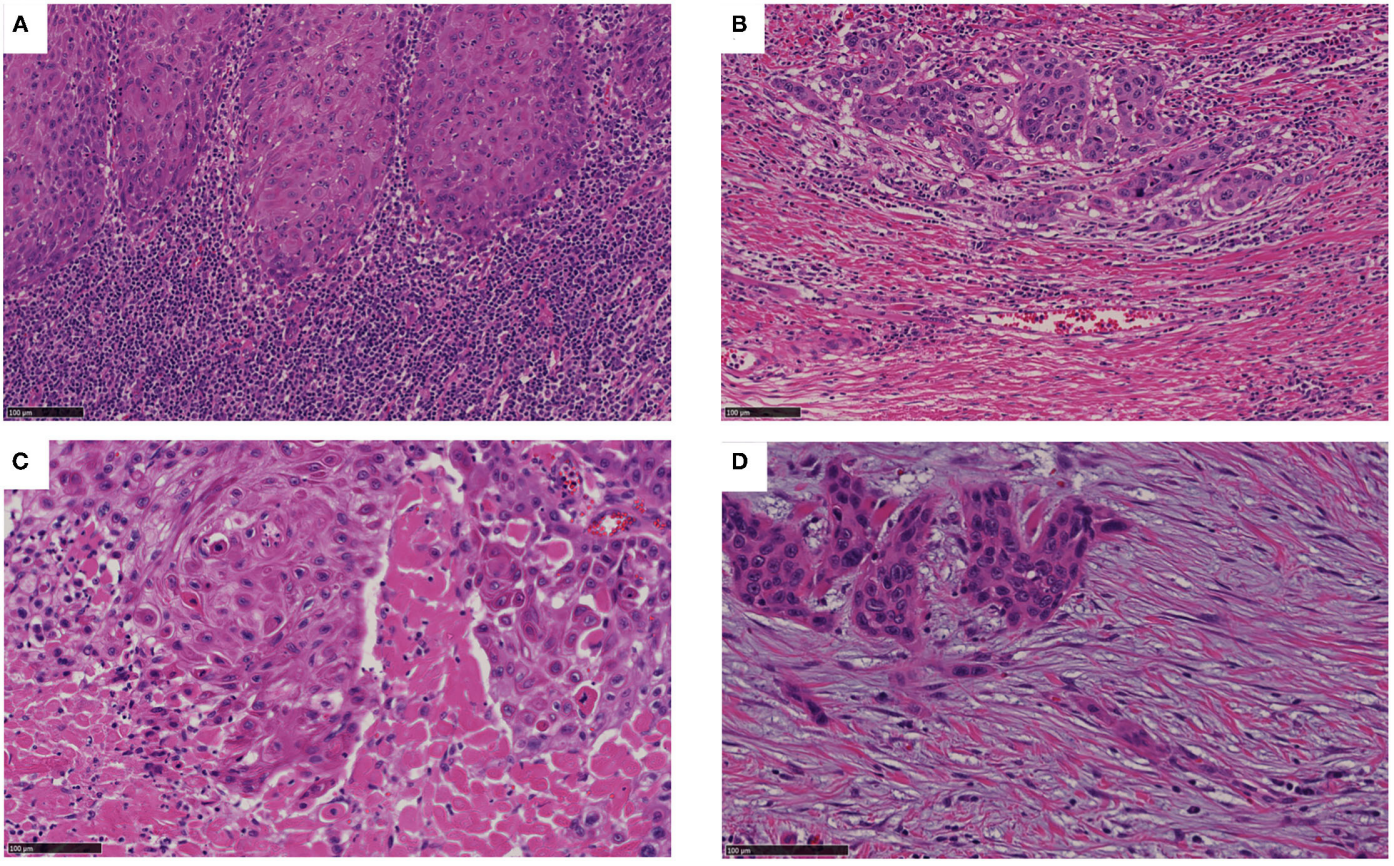
Categorization of SP. **(A)** The inflammatory type involves an inconspicuous desmoplastic reaction and lymphocytic infiltration into the stroma (as shown). **(B)** The mature type involves a fibrotic stroma lacking keloid-like collagen and a myxoid stroma. **(C)** The intermediate type can be identified based on the presence of keloid-like collagen (i.e., fragmented broad bands of collagen exhibiting brightly eosinophilic hyalinization, similar to those seen in a keloid). **(D)** The immature type can be identified based on abundant amorphous extracellular matrix material in a myxoid stroma. Bar: 100 μm.

According to the criteria described in the Methods, we classified the SP at the invasive front of OSCC into four types; i.e., into the immune/inflammatory (Figure 2A), mature (Figure 2B), intermediate (Figure 2C), and immature (Figure 2D) types. Overall, 84, 78, 14, and 56 cases were classified as belonging to the immune/inflammatory, intermediate, mature, and immature type, respectively. Since only 14 cases were classified as the mature type, the intermediate and mature types were combined into the intermediate/mature type according to the method described by Ao et al. (14).

The correlations between the SP category and clinicopathological characteristics are shown in Table 1. There were significant correlations between the SP category and the YK grade or other conventional pathological prognostic and treatment factors. For each conventional factor, the immature type was associated with high-risk categories, and the immune/inflammatory type was associated with low-risk categories.

**Table 1:** Correlations between the SP and clinicopathological factors.

**Factors**	**Inflammatory**	**Int + Mature**	**Immature**	***p* value**
	**(*n =* 84)**	**(*n =* 92)**	**(*n =* 56)**	
**Age**, ***n*** **(%)**				
Over 50	67 (79.8)	77 (83.7)	42 (75)	0.4339
Under 50	17 (20.2)	15 (16.3)	14 (25)	
**Gender**, ***n*** **(%)**			
Male	45 (53.6)	53 (57.6)	29 (51.8)	0.7599
Female	39 (46.4)	39 (42.4)	27 (48.2)	
**Location**, ***n*** **(%)**			
Tongue	55 (65.5)	54 (58.7)	35 (62.5)	0.6494
Others	29 (34.5)	38 (41.3)	21 (37.5)	
**Histological grade**, ***n*** **(%)**				
1,2	84 (100)	89 (96.7)	46 (82.1)	<0.0001
3	0	3 (3.3)	10 (17.9)	
**pT**, ***n*** **(%)**				
pT1,2	64 (76.2)	68 (73.9)	27 (48.2)	0.0008
pT3,4	20 (23.8)	24 (26.1)	29 (51.8)	
**Stage**, ***n*** **(%)**			
I,II	64 (76.2)	60 (65.2)	24 (42.9)	0.0003
III, IV	20 (23.8)	32 (34.8)	32 (57.1)	
**Ly/v invasion**, ***n*** **(%)**			
Negative	48 (57.1)	42 (45.7)	8 (14.3)	<0.0001
Positive	36 (42.9)	50 (54.3)	48 (85.7)	
**Neu invasion**, ***n*** **(%)**			
Negative	71 (84.5)	68 (73.9)	28 (50)	<0.0001
Positive	13 (15.5)	24 (26.1)	28 (50)	
**LN metastasis**, ***n*** **(%)**				
Negative	76 (90.5)	68 (73.9)	29 (51.8)	<0.0001
Positive	8 (9.5)	24 (26.1)	27 (48.2)	
**ENE**, ***n*** **(%)**				
Negative	79 (94.0)	77 (83.7)	40 (71.5)	0.0014
Positive	5 (6.0)	15 (16.3)	16 (28.5)	
**YK**, ***n*** **(%)**				
1,2,3	66 (78.6)	50 (54.3)	7 (12.5)	<0.0001
4C, 4D	18 (21.4)	42 (45.7)	49 (87.5)	
**Postoperative radiotherapy**, ***n*** **(%)**				
No	70 (83.3)	74 (80.4)	37 (66.1)	0.0718
Yes	14 (16.7)	18 (19.6)	19 (33.9)	
**Postoperative chemotherapy**, ***n*** **(%)**				
No	74 (88.1)	81 (88.0)	39 (69.6)	0.0052
Yes	10 (11.9)	11 (12.0)	17 (30.4)	
**Lymph node dissection**, ***n*** **(%)**				
No	54 (64.3)	50 (54.3)	17 (30.4)	0.0004
Yes	30 (35.7)	42 (45.7)	39 (69.6)	
**Reconstruction**, ***n*** **(%)**				
No	55 (65.5)	53 (57.6)	18 (32.1)	0.0004
Yes	29 (34.5)	39 (42.4)	38 (67.9)	

*LN, lymph node; ENE, extra nodal extension*.

### Correlations Between the SP Category and the Prognosis of OSCC

**Figure 3:**
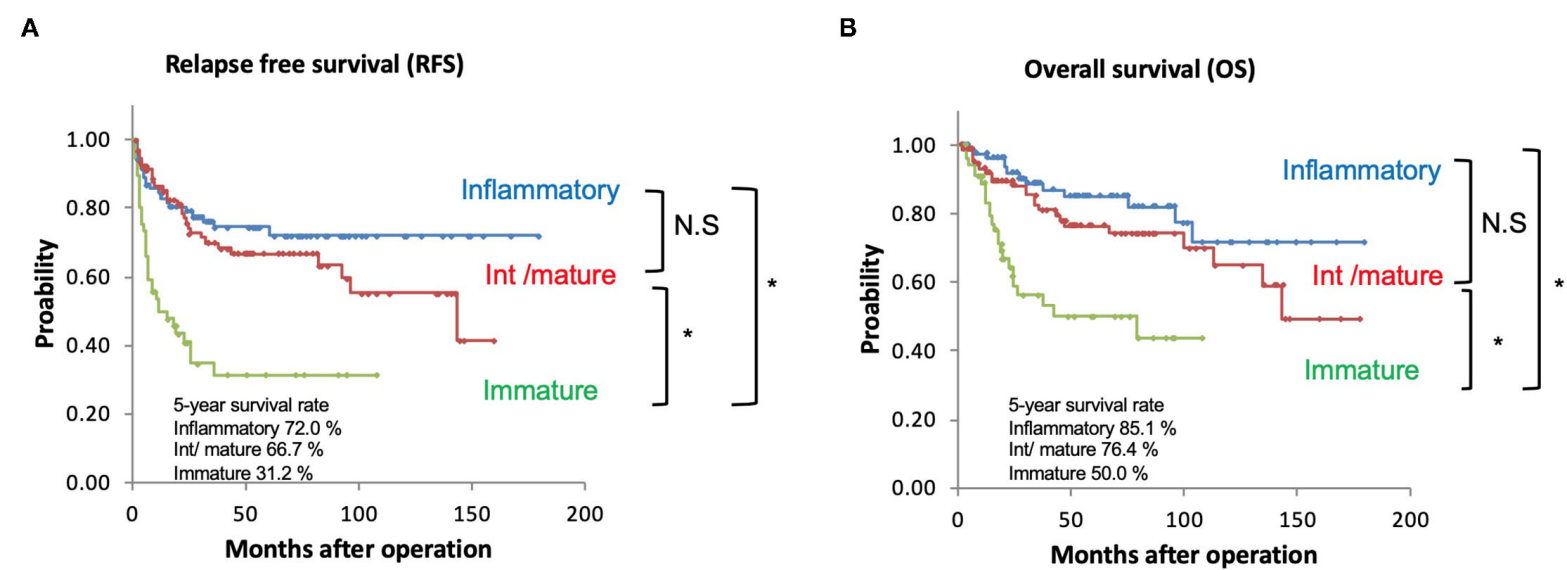
**(A)** Kaplan-Meier curves of recurrence-free survival based on the SP. **(B)** Kaplan-Meier curves of overall survival based on the SP. *p < 0.001; NS, not significant.

The 5-year RFS rate was 72.0% for the immune/inflammatory type, 66.7% for the intermediate/mature type, and 31.2% for the immature type. The SP category was significantly associated with the RFS rate (immune/inflammatory vs. immature, intermediate/mature vs. immature, both *p* < 0.001) (Figure 3A).

The 5-year OS rate was 85.1% for the immune/inflammatory type, 76.4% for the intermediate/mature type, and 50.0% for the immature type. The SP category was significantly associated with the OS rate (immune/inflammatory vs. immature, intermediate/ mature vs. immature, both *p* < 0.01) (Figure 3B).

Next, we performed univariate and multivariate analyses using the Cox proportional hazards model to identify parameters that are associated with RFS or OS in patients with OSCC.

With regard to RFS, when the immune/inflammatory group was used as a standard, the HR of the intermediate/mature and immature groups were 1.3898 (95% CI, 0.7954–2.4285, *p* = 0.2477) and 3.6503 (95% CI, 2.1059–6.3274, *p* < 0.001), respectively (Table 2). In the multivariate analysis, the lymph node metastasis and SP were identified as significant predictors of prognosis, whereas the YK grade was only marginally associated with poor prognosis (Table 2).

**Table 2:** Univariate and multivariate analyses of relapse-free survival.

**Factors**	**Univariate analysis**			**Multivariate analysis**		
	**HR**	**95 %CI**	***p* value**	**HR**	**95 %CI**	***p* value**
**Age**						
Under 60	1					
Over 60	1.0653	0.6400–1.7734	0.8078			
**Gender**						
Female	1					
Male	1.1260	0.7376–1.7191	0.5824			
**Location**						
Tongue	1					
Others	1.1717	0.7580–1.8114	0.4758			
**Histological type**						
Grade 1,2	1			1		
Grade 3	2.8191	1.8348–4.3314	*p* <0.001	1.6422	0.8891–3.0333	0.1131
**pT**						
1, 2	1					
3, 4	1.4526	0.8924–2.3644	0.1331			
**Stage**						
I, II	1			1		
III, IV	2.0699	1.3463–3.1825	*p* <0.001	0.7380	0.3228–1.6871	0.4714
**Ly/v invasion**						
Negative	1			1		
Positive	2.5944	1.6041–4.1959	*p* <0.001	1.0791	0.4914–2.3696	0.8496
**Neural invasion**						
Negative	1			1		
Positive	3.5613	2.3128–5.4838	*p* <0.001	1.7354	0.9061–3.3239	0.0964
**LN metastasis**						
Negative	1			1		
Positive	2.9546	1.9035–4.5860	*p* <0.001	2.5734	1.1915–5.5582	0.0161
**ENE**						
Negative	1			1		
Positive	3.9353	2.4298–6.3736	*p* <0.001	1.2089	0.4352–3.3584	0.7159
**YK**						
1, 2, 3	1			1		
4C, 4D	3.0162	1.9161–4.7481	*p* <0.001	2.1613	0.9431–4.9529	0.0685
**Pattern**						
Inf	1			1		
Int + Mature	1.3898	0.7954–2.4285	0.2477	1.1131	0.5992–2.0676	0.7345
Immature	3.6503	2.1059–6.3274	*p* <0.001	2.7126	1.0924–6.7362	0.0315
**Postoperative radiotherapy**						
No	1			1		
Yes	1.7862	1.1192–2.8507	0.015	0.8484	0.3825–1.8818	0.6859
**Postoperative chemotherapy**
No	1			1		
Yes	2.255	1.3962–3.6419	*p* <0.001	0.8304	0.4024–1.7132	0.6149
**Lymph node dissection**
No	1					
Yes	1.2056	0.7886–1.8431	0.3880			
Reconstruction^*^
No	1					
Yes	1.2025	0.7864–1.8389	0.3947			

* *The methods of surgical reconstruction were as follows: the fibula flap, 13 cases; the rectus abdominis musculocutaneous (RAMC) flap, 8 cases; the grolin flap, 16 cases; the anterolateral thigh (ALT) type, 16 cases; the pectoralis major musculocutaneous (PMMC) flap, 1 case; the free RFA flap, 7 cases; the latissimus dorsi musculocutaneous (LDMC) flap, 1 case; the platysma musculocutaneous flap, 1 case; and unknown, 2 cases. No associations were observed between the methods of surgical reconstruction and the stromal pattern*.

*LN, lymph node; ENE, extra nodal extension*.

As for OS, when the immune/inflammatory group was used as a standard, the HR of the intermediate/mature and immature groups were 1.6191 (95% CI, 0.8199–3.1974, *p* = 0.1652) and 2.0830 (95% CI, 1.4711–2.9495, *p* <0.001), respectively (Table 3). In the multivariate analysis, only neural invasion, lymph node metastasis, and the SP were identified as significant predictors of prognosis (Table 3).

**Table 3:** Univariate and multivariate analyses of overall survival.

**Factors**	**Univariate analysis**			**Multivariate analysis**		
	**HR**	**95 %CI**	***p* value**	**HR**	**95 %CI**	***p* value**
**Age**						
Under 60	1					
Over 60	1.2612	0.7026–2.2641	0.4369			
**Gender**						
Female	1					
Male	1.5235	0.9159–2.5343	0.1049			
**Location**						
Tongue	1					
Others	1.6502	0.9906–2.7491	0.0544			
**Histological type**						
Grade 1, 2	1			1		
Grade 3	2.0795	1.2338–3.5047	0.0060	0.8064	0.3635–1.7888	0.5965
**pT**						
1, 2	1			1		
3, 4	3.0103	1.7668–5.1289	*p* <0.001	0.5992	0.2216–1.6199	0.3128
**Stage**						
I,II	1			1		
III,IV	3.7338	2.2133–6.2989	*p* <0.001	3.2113	0.8172–12.6183	0.0947
**Ly/v invasion**						
Negative	1			1		
Positive	2.5088	1.4144–4.4502	0.0017	0.5354	0.1666–1.7199	0.5354
**Neural invasion**						
Negative	1			1		
Positive	4.2947	2.5410–7.2586	*p* <0.001	3.3084	1.2166–8.9970	0.0191
**LN metastasis**
Negative	1			1		
Positive	3.9423	2.3279–6.6762	*p* <0.001	8.4807	1.8632–38.6018	0.0057
**ENE**						
Negative	1			1		
Positive	5.4225	3.0457–9.6540	*p* <0.001	0.8429	0.2305–3.0819	0.7961
**YK**						
1, 2, 3	1			1		
4C, 4D	3.0162	1.9161–4.7481	*p* <0.001	0.4448	0.1432–1.3819	0.1613
**Pattern**						
Inf	1			1		
Int + Mature	1.3898	0.7954–2.4285	0.2477	1.3814	0.6531–2.9217	0.3979
Immature	3.6503	2.1059–6.3274	*p* <0.001	3.3203	1.0180–10.8288	0.0466
**Postoperative radiotherapy**
No	1			1		
Yes	3.1249	1.8604–5.2490	*p* <0.001	1.0701	0.3826–2.9931	0.8973
**Postoperative chemotherapy**
No	1			1		
Yes	2.3301	1.3260–4.0945	0.0033	1.1776	0.5163–2.6856	0.6976
**Lymph node dissection**
No	1			1		
Yes	2.0136	1.1946–3.3944	0.0086	0.3074	0.0406–2.3266	0.2533
Reconstruction^*^
No	1			1		
Yes	2.0369	1.2128–3.4208	0.0072	1.3770	0.1903–9.9626	0.7514

* *The methods of surgical reconstruction were as follows: the fibula flap, 13 cases; the rectus abdominis musculocutaneous (RAMC) flap, 8 cases; the grolin flap, 16 cases; the anterolateral thigh (ALT) type, 16 cases; the pectoralis major musculocutaneous (PMMC) flap, 1 case; the free RFA flap, 7 cases; the latissimus dorsi musculocutaneous (LDMC) flap, 1 case; the platysma musculocutaneous flap, 1 case; and unknown, 2 cases. No associations were observed between the methods of surgical reconstruction and the stromal pattern*.

*LN, lymph node; ENE, extra nodal extension*.

## Discussion

A desmoplastic reaction (DR) is defined by the growth of fibrous or connective tissue at sites of stromal invasion by cancer (15). The presence of a DR has been reported to be associated with poor outcomes in several types of cancer, such as those of the colon, pancreas, neuroendocrine organs, and skin (11, 16–18). On the basis of the distinctive histological features of hematoxylin and eosin (HE)-stained desmoplastic stromal tissue, Ueno et al. divided the DR of colon cancer into three groups; i.e., into mature, intermediate, and immature types (19). This DR categorization system provided prognostic information about colorectal cancer patients; i.e., the 5-year survival rate was 80% for the mature type, 55% for the intermediate type, and 27% for the immature type (19).

In addition to DR, immune/inflammatory cells, such as granulocytes, lymphocytes, and macrophages, also play a key role in the TME (20). These cells are involved in various immune responses and defensive activities against tumor cells. Therefore, the immune system in the TME may influence patient prognosis and the treatment response (20). Previous studies have shown that tumor-infiltrating immune cells are closely associated with the prognoses of colorectal, renal, and oral cancers (21–23).

In the present study, we classified the SP of OSCC into immune/inflammatory, mature, intermediate, and immature types, based on the findings of HE-stained slides. We examined the utility of this new SP classification by analyzing its clinicopathological correlations and prognostic value.

One main finding of this study was that the immature type was associated with aggressive tumor features, such as poor differentiation (grade 3), lymphovascular or neural invasion, and an infiltrative invasive pattern (YK-4C or YK-4D), and patient survival.

A myxoid stroma has also been reported to be associated with aggressive tumor histological features and a poor prognosis in colorectal cancer (11, 24, 25). Ueno et al. classified the DR of colorectal cancer into 3 types (mature, intermediate, and immature). The immature type was found to be associated with the dedifferentiation of tumor cells; hypovascularity; a restricted distribution of immune cells; and abundant tumor-supporting extracellular components, including fibronectin and tenacin (24, 25). The prognostic power of this DR categorization system for stratifying 5-year RFS was greater than that of any other conventional prognostic factor, such as the TNM stage, venous invasion, or tumor grade (11, 24).

A DR may represent the histological consequences of ECM remodeling generated by CAF (15). In the TME, CAF play a critical role in mediating the epithelial-mesenchymal transition (EMT) of carcinoma cells (6). EMT is a complex and reversible biological process through which epithelial tumors change from a polar, adhesive phenotype to a mesenchymal phenotype characterized by increased cell migration, invasive potential, cytoskeletal remodeling, and resistance to apoptosis (26). Ueno et al. speculated that keloid-like collagen and a myxoid stroma may represent histological features that are associated with the induction of EMT in tumor cells (27).

In this study, we modified the DR classification described by Ueno et al. by adding the immune/inflammatory type, which is characterized by diffuse immune/inflammatory cell infiltration and inconspicuous desmoplasia at the invasive front, as a new category. We showed that the immune/inflammatory type was associated with shallow invasion, early stage disease, the absence of lymphovascular or neural invasion, and expansive-type invasion. These results are in line with the findings obtained for cutaneous SCC (18). In cutaneous SCC, the presence of a myxoid stroma was found to be associated with a lack of inflammatory and immune reactions, perineural invasion, tumor depth, and metastatic risk (18). The authors speculated that the absence of an immune response in an immature myxoid stroma facilitates the metastatic process (18). In our study, immune/inflammatory cells were largely lymphoid cells, as far as we could identify on HE-stained sections. As infiltration of immune cells may represent host reaction to combat malignancy, it would make sense that the immune/inflammatory type was associated with favorable histologic features and better prognosis. Results supporting this notion have recently been reported by Troniano et al. These authors demonstrated that paucity of immune reaction predicted poor prognosis in tongue squamous cell carcinoma (28).

The existence of an immune/inflammatory subtype is supported by recent studies of carcinoma of the lung, breast, or bladder based on RNA sequencing or proteomic data (21–23, 29–31). In breast cancer, an immunohistochemistry-based classification exhibited substantial agreement with an mRNA expression-based classification (30). The immunomodulatory type, which is characterized by the infiltration of CD8-positive T cells into the tumor parenchyma, showed a better prognosis than the other subtypes and relatively high expression of immune checkpoint molecules, such as cytotoxic T-lymphocyte-associated protein 4 (CTLA4), programmed death ligand 1 (PD-L1), indoleamine 2, 3-dioxygenase 1 (IDO1), and programmed cell death protein 1 (PD-1) (30). In bladder cancer, the inflamed type demonstrated the highest levels of many immune cell types, including cytotoxic lymphocytes, T cells, and the immune regulators nuclear factor of activated T-cells, cytoplasmic 2 (NFATC2) and signal transducer and activator of transcription 4 (STAT4), while the MYC-driven subtypes, including the Myc type and early type, exhibited repressed immune responses and worse prognoses than the inflamed type (31).

This study had several limitations. First, the number of cases was smaller than those in previous studies of colon cancer (11). Second, immature type was strongly correlated with advanced cases, neural invasion, and YK 4C, 4D. Although our multivariate analysis indicated an independent predictive value of stromal pattern in our cohort, the close associations among multiple factors may make it difficult to firmly establish that SP is a truely independent prognostic factor. A larger number of cases from multiple facilities might be required to confirm the current findings. Third, the precise molecular mechanisms by which the SP contributes to the TME in OSCC remain unknown.

The molecules involved in the interactions between tumor cells and the adjacent fibroblasts may include Wnt/beta-catenin, transforming growth factor beta, and C-X-C chemokine receptor type 4 (CXCR4) (24, 27, 32–34). Recently, Ueno et al. showed that both the mRNA and protein expression levels of periostin were high in the immature type of colon cancer (35). Periostin has been shown to be an important regulator of bone and tooth formation and maintenance and EMT (36). Ogawa et al. identified three distinct stromal types that influence survival, immunity, and molecular features in pancreatic cancer (37). Further studies are warranted to elucidate the molecular mechanisms underlying each type of SP and their effects on the TME.

In conclusion, in OSCC the SP is associated with prognosis and may predict tumor aggressiveness. Our SP categorization method may be an excellent tool for aiding decision-making regarding treatment options in precision medicine and provide a histological basis for further clinical and molecular research.

## Data Availability Statement

The raw data supporting the conclusions of this article will be made available by the authors, without undue reservation.

## Ethics Statement

The studies involving human participants were reviewed and approved by Jichi Medical University. The Ethics Committee waived the requirement of written informed consent for participation. Written informed consent was not obtained from the individual(s) for the publication of any potentially identifiable images or data included in this article. There are no potentially identifiable data or images are presented in the article.

## Author Contributions

YA: conceptualization, investigation, and writing original draft. AK and TM: prepared the tissue samples and validated the data. MH: prepared the tissue samples, validated the data, and performed histological evaluation. DM: validated the data. NF, HN, and YM: collected the clinical records. TN: resources, supervision, writing review, and editing. All authors critically reviewed and approved the manuscript.

## Funding

This work was supported in part by Japan Society for the Promotion of Science KAKENHI Grant Number 19K10094 (to YA).

## Conflict of Interest

The authors declare that the research was conducted in the absence of any commercial or financial relationships that could be construed as a potential conflict of interest.

## Publisher's Note

All claims expressed in this article are solely those of the authors and do not necessarily represent those of their affiliated organizations, or those of the publisher, the editors and the reviewers. Any product that may be evaluated in this article, or claim that may be made by its manufacturer, is not guaranteed or endorsed by the publisher.
